# Seasonality of moisture supplies to precipitation over the Third Pole: a stable water isotopic perspective

**DOI:** 10.1038/s41598-020-71949-0

**Published:** 2020-09-14

**Authors:** Xiaoxin Yang, Tandong Yao

**Affiliations:** 1 Laboratory of Tibetan Environmental Changes and Land Surface Processes, Institute of Tibetan Plateau Research, Chinese Academy of Sciences, Beijing, 100101 China; 2 Center for Excellence in Tibetan Plateau Earth Sciences, Chinese Academy of Sciences, Beijing, 100101 China

**Keywords:** Atmospheric dynamics, Climate sciences

## Abstract

This study integrated isotopic composition in precipitation at 50 stations on and around the Tibetan Plateau (TP) and demonstrated the distinct seasonality of isotopic composition in precipitation across the study period. The potential effect of water vapor isotopes on precipitation isotopes is studied by comparing the station precipitation data with extensive isotopic patterns in atmospheric water vapor, revealing the close linkage between the two. The analysis of contemporary water vapor transport and potential helps confirm the different mechanisms behind precipitation isotopic compositions in different areas, as the southern TP is more closely related to large-scale atmospheric circulation such as local Hadley and summer monsoon circulations during other seasons than winter, while the northern TP is subject to the westerly prevalence and advective moisture supply and precipitation processes. The new data presented in this manuscript also enrich the current dataset for the study of precipitation isotopes in this region and together provide a valuable database for verification of the isotope-integrated general circulation model and explanation of related physical processes.

## Introduction

The Tibetan Plateau (TP) and its surroundings, known as the Third Pole on earth, accommodates the largest ice mass outside the Arctic and Antarctica. Under global warming, glaciers in the region are undergoing overall melting with elevation-dependent warming^1,2^ and heterogeneous variation with the westerly-monsoon interactions^3^. Precipitation input as a crucial term in the water budget on the TP, on the other hand, is difficult to evaluate given its complex topography, large spatial expanse and meteorological factors (such as wind blowing, precipitation types etc.) affecting precipitation accuracy in mountainous regions as on the TP (e.g.,^4–6^). Numerous efforts have been done to shed light on water supplies to various parts of the TP^7–9^, but an overall picture of moisture supply across the TP with seasons is still lacking. Yet a complete understanding of water supplies seasonality to various parts of the TP will facilitate a comprehensive understanding of the interaction between the summer Indian monsoon and prevailing winter westerlies, which controls the seasonal shifts of precipitation amounts and sources^10^ over the TP known as Asian water tower^11^, and helps project, given an accurate understanding of the controlling mechanism in each sector, future hydrological scenarios.

In this sense, isotopic compositions in precipitation work as tracers and provide one method for observationally determining atmospheric circulation impacting the TP. So far, water stable isotopes in precipitation have demonstrated wide applications to earth sciences on the TP, having been used to detect moisture source and intensity of related atmospheric circulations^12–16^, reflect the influences of local and/or large-scale atmospheric circulation processes on precipitation that pertains closely to the water cycle^17–21^, transcend time to link modern variation features with climate significances in paleoproxies^5,12,22–26^ and inform of the plateau uplift history with their imprints on rock records^27,28^. Various studies on water stable isotope compositions have been conducted in the southern, southeastern, northeastern and northwestern parts of TP^29–32^, revealing the dependency of δ^18^O in precipitation on moisture sources^17,33^, relative humidity^22^, cloudiness^34^, and integrated convective activity along upstream air mass trajectories^17,30,35–37^. Those studies highlighted the spatial heterogeneity of precipitation and its water stable isotopic compositions across the TP, which pertains to atmospheric circulations. An overall picture of moisture sources and associated storm activity along air mass trajectories would be conducive to a better understanding of controlling factors over isotopic variations at the seasonal scale^37^, and thus finally contributes to a better understanding of the westerlies-monsoon interactions under current climate scenarios.

This paper intends to pool stable isotopic compositions in precipitation at as many stations as possible from our observation network plus the Global Network on Isotopes in Precipitation (GNIP) to give a spatial overview of the seasonality of isotopic variation and to apply tropospheric emission spectrometry (TES) vapor data together with high-resolution atmospheric reanalysis data to explore possible mechanisms responsible for the spatial heterogeneity in different seasons. The launch of TES allows the capture of a holistic picture of HDO (i.e., water with heavier hydrogen isotopologue) and H_2_O (i.e., most water component with light hydrogen isotopologue) composition throughout the globe every two days^38^. The estimated HDO/H_2_O can profile the HDO/H_2_O ratio between 925 and 450 hPa in the tropics^39,40^. Consideration of the ratio together with ground observations of precipitation will facilitate previous studies that focused solely on stable isotopes in precipitation. Thus, mobile and three-dimensional monitoring of moisture transport to the TP is possible. Through studying the seasonality in the spatial distribution of long-term δ^18^O climatology, this research intends to provide an overall picture of precipitation stable isotopic signals with seasons, identify possible moisture sources, and water mass transport routes and processes responsible for the spatial distinction, and thereupon shed light on the seasonally distinct impacts of large-scale atmospheric circulation on regional precipitation over the TP.

## Results

**Figure 1:**
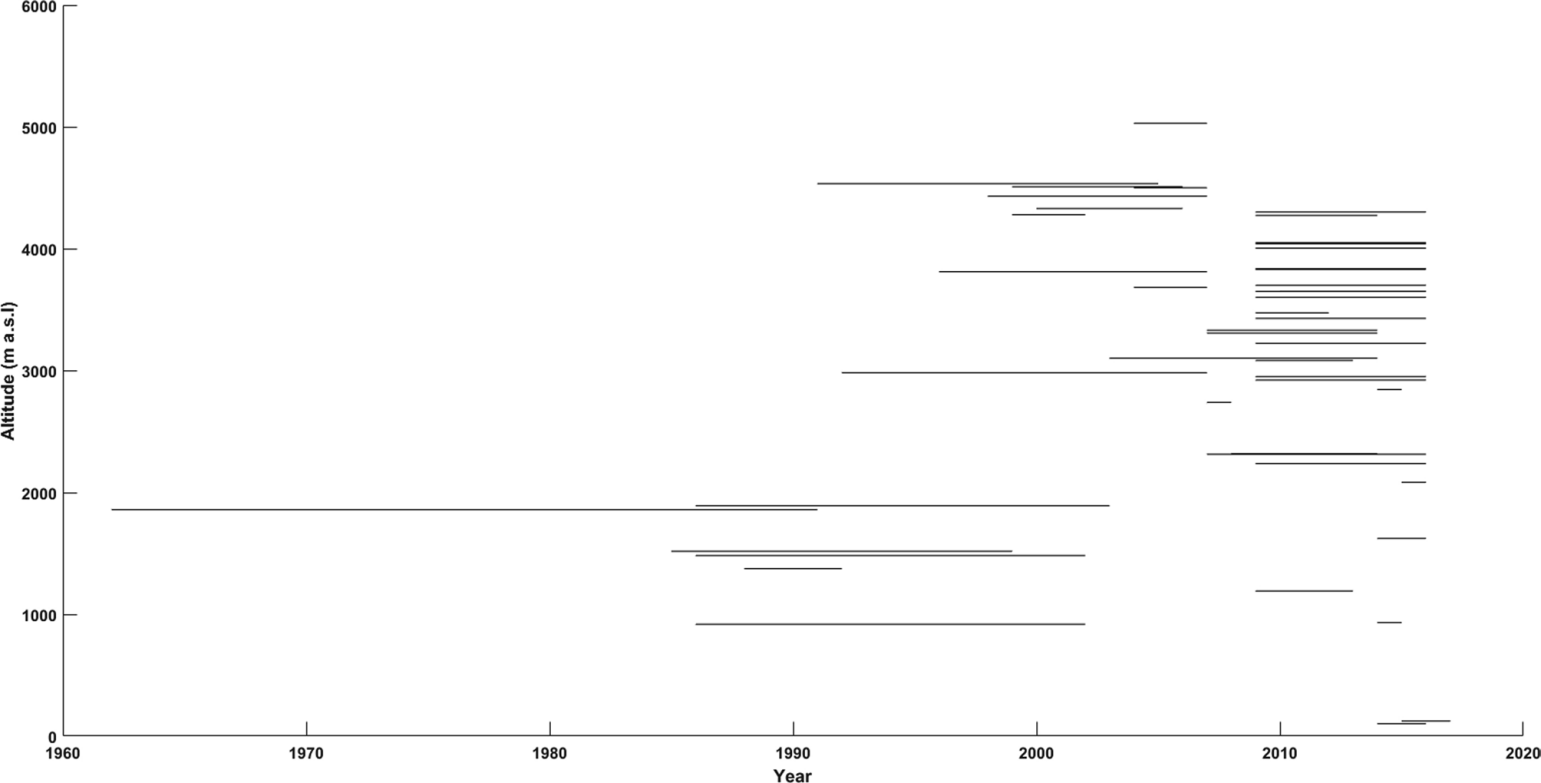
Duration of all station data used in this study.

We studied 50 stations on and around the TP, having integrated ground stations set up by the Third Pole Environment with our existing stations and the GNIP dataset (https://www.iaea.org/services/networks/gnip) (Fig. 1). Details of the stations, including their geographical locations, altitudes, durations and references, are presented in Table 1. The durations of the station data range from one year (at Lobuche, Nepal Himalaya) to 30 years (at Kabul, Afghanistan). The total time period of the dataset across the TP covers over 56 years (1962–2018).

**Table 1:** Details of stations whose data are used in this study.

Stid	Stnm	Lat (N)	Lon (E)	Alt (m)	Start_mon	End_mon	No. mons	References
1	Zayu	28.67	97.47	2,314	2007-7	2016-12	106	This study
2	Lulang	29.77	94.73	3,330	2007-1	2014-6	79	This study
3	Gengzhang	29.73	94.15	3,082	2009-5	2013-9	22	This study
4	Baheqiao	29.83	93.67	3,223	2009-5	2016-10	69	This study
5	Larze	29.12	87.57	4,004	2009-5	2016-9	48	This study
6	Yangcun	29.28	91.88	3,600	2009-5	2016-10	59	This study
7	Nugesha	29.33	89.72	3,700	2009-5	2016-10	46	This study
8	Jiangzi	28.94	89.63	4,040	2009-5	2016-10	59	This study
9	Rikaze	29.28	88.82	3,836	2009-6	2016-9	44	This study
10	Lhasa	29.65	91.20	3,649	2009-5	2016-10	65	This study
11	Nuxia	29.47	94.65	2,920	2009-6	2016-10	63	This study
12	Gongbujiangda	29.92	93.25	3,427	2009-5	2016-10	49	This study
13	Pangduo	30.18	91.33	4,048	2009-5	2016-10	67	This study
14	Yangbajing	30.09	90.54	4,300	2009-5	2016-10	61	This study
15	Tangjia	29.88	91.77	3,830	2009-5	2016-12	64	This study
16	Milin	29.18	94.13	2,950	2009-6	2016-9	54	This study
17	Taxkorgan	37.77	75.27	3,100	2003-9	2014-10	77	This study
18	Bulunkou	38.65	74.97	3,306	2007-7	2014-11	82	This study
19	Keleke	38.78	75.32	2,320	2008-4	2014-11	59	This study
20	Guide	36.03	101.43	2,238	2009-5	2016-5	66	This study
21	Mado	34.92	98.22	4,273	2009-5	2014-7	44	This study
22	Maqu	34.00	102.08	3,473	2009-5	2012-3	34	This study
23	Panzhihua	26.58	101.72	1,191	2009-4	2013-5	38	This study
24	Kuerle	41.75	86.13	933	2014-5	2015-9	13	This study
25	Delingha	37.37	97.37	2,981	1992-2	2007-9	133	13
26	Nagqu	31.48	92.07	4,508	1999-9	2006-10	70	13
27	Gaize	32.30	84.07	4,430	1998-7	2005-9	46	13
28	Shiqianhe	32.50	80.08	4,278	1999-2	2002-8	24	13
29	Tingri	28.65	87.12	4,330	2000-6	2006-8	21	13
30	Nylam	28.18	85.97	3,810	1996-8	2007-9	91	13
31	Tuotuohe	34.22	92.43	4,533	1991-9	2005-9	104	13
32	Baidi	29.12	90.43	4,430	2004-1	2007-9	22	13
33	Bomi	29.87	95.77	2,737	2007-10	2008-11	13	13
34	Dui	28.58	90.53	5,030	2004-2	2007-10	24	13
35	Zhangmu	27.98	85.98	2,239	2005-7	2005-10	4	13
36	Wengguo	28.90	90.35	4,500	2004-4	2007-10	14	13
37	Kunming	25.01	102.68	1892	1986-1	2003-12	201	GNIP
38	Hetian	37.08	79.56	1,375	1988-2	1992-12	59	GNIP
39	Lanzhou	36.05	103.88	1517	1985-7	1999-12	77	GNIP
40	Zhangye	38.93	100.43	1,483	1986-7	2002-12	110	GNIP
41	Urumqi	43.78	87.62	918	1986-1	2002-12	144	GNIP
42	Kabul	34.67	69.08	1,860	1962-1	1991-12	359	GNIP
43	Mutztag	38.23	75.01	3,650	2010-7	2016-10	30	This study
44	Yushu	33.02	97.02	3,682	2004-1	2007-12	47	This study
45	Naintal	29.40	79.45	2084	2015-1	2016-9	20	This study
46	Luknow	26.85	80.95	123	2015-1	2017-3	22	This study
47	Siraha	26.65	86.22	102	2014-5	2016-10	23	41
48	Diktel	27.22	86.80	1623	2014-5	2016-10	27	41
49	Lukla	27.68	86.73	2,843	2014-5	2015-10	15	41
50	Lobuche	27.95	86.81	5,050	2016-5	2016-9	5	41

**Figure 2:**
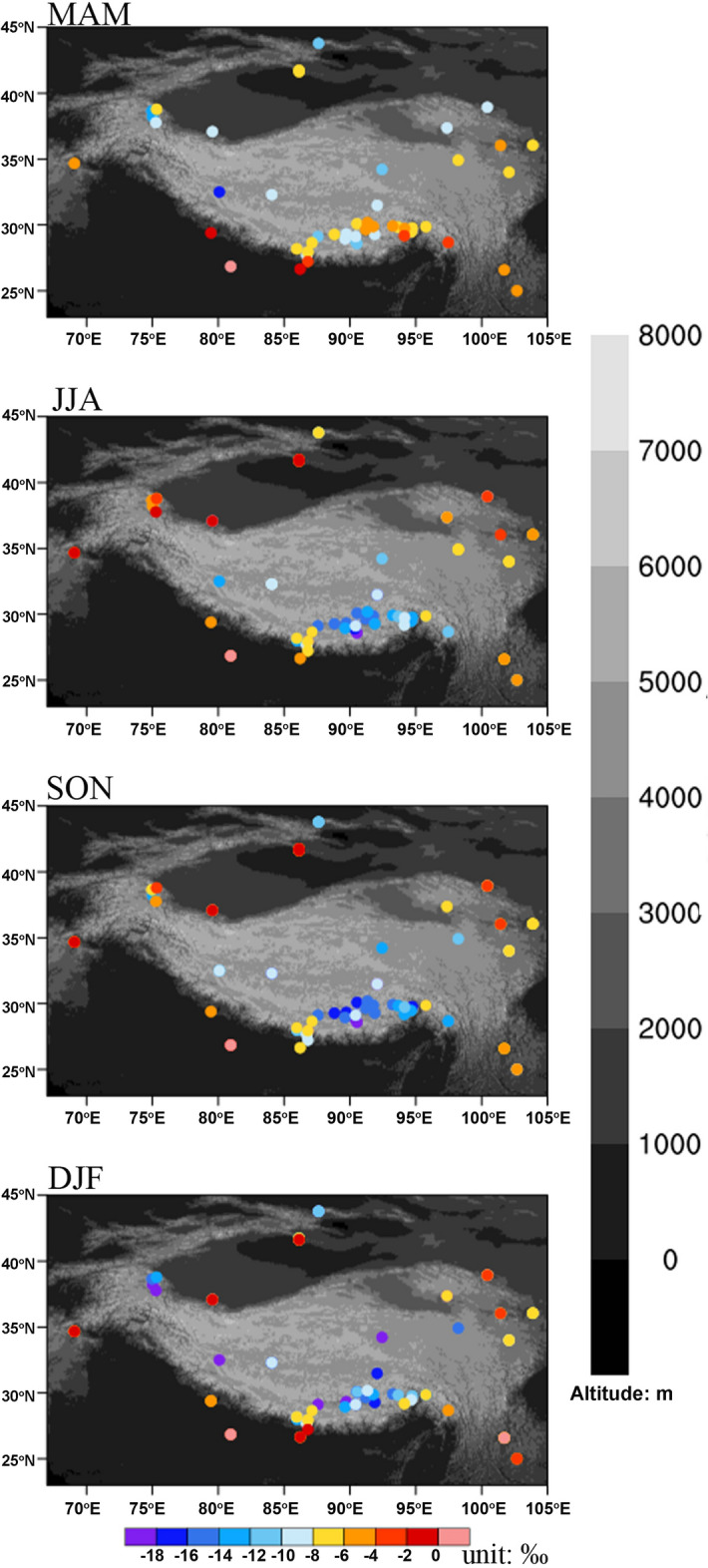
Seasonality of δ^18^O in precipitation (δ^18^O_p_) over the Tibetan Plateau and its adjacent region. From the top down, the panels represent the spatial distribution of δ^18^O_p_ in MAM, JJA, SON and DJF. The colours of the dots indicate isotopic values, with warmer colours denoting comparatively high values, while colder colours denote low values. The background colour denotes the altitude in metres. The figure is plotted using the NCAR Command Language (Version 6.6.2) [Software]. (2019). (Boulder, Colorado: UCAR/NCAR/CISL/TDD. https://dx.doi.org/10.5065/D6WD3XH5).

The spatial isotopic composition in precipitation demonstrates interesting features surrounding the plateau. As is shown in Fig. 2, in regions to the west, south and east of the plateau, the isotopic composition in precipitation is generally enriched without clear seasonal distinction throughout the year compared to that on the plateau. Similarly, the isotopic composition in precipitation in the interior plateau shows overall depletion without seasonal distinction throughout the year. In comparison, isotopic compositions in precipitation on the edge of the plateau show distinct seasonality and northwest-southeast contrast, featuring high values during months from March to May (MAM) in the southeastern part of the TP followed by low values in the other three seasons, while in the northwestern part, low values during MAM and DJF (i.e., months from December to next February) are in contrast with high values in the other two seasons (Fig. 2).

The contrast between the northwest and southeast TP can be further shown by the difference in the median values of monthly δ^18^O_p_ (from -18‰ in the north to -4‰ in the south during January-March to less difference between the north and south during April-June, then to − 10‰ in the north and − 15‰ in the south during July–August, and finally returning to a low value in the north, − 15‰, and a high value in the south, − 7‰, during November–December). There is a clear coexistence of north/west high and south/east low values during June-October, with the difference ranging from 1‰ (2‰) to 4‰ (13.5‰) in the zonal (meridional) distribution. A contrary spatial distribution pattern is clearly noted during the winter months (December-March), with the largest east–west (by 9‰) and north–south (by 14‰) differences both shown in January. The heterogeneity of isotopic seasonality on the northwestern and southeastern corners of the TP may be attributed to the responses/interactions of/between large-scale atmospheric circulation to/and high topography, which will be discussed below in detail.

## Discussion

### Precipitation seasonality and mechanisms

**Figure 3:**
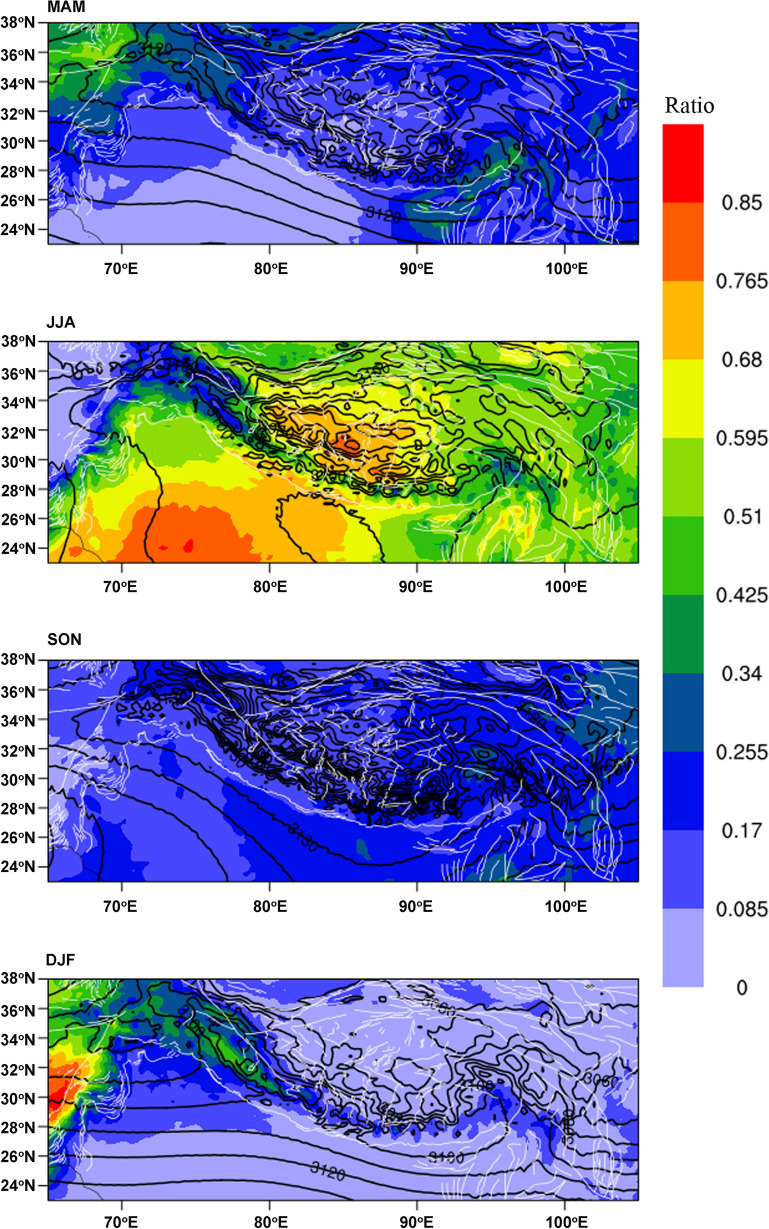
Precipitation seasonality over the TP and its surroundings. Ratios of seasonal to annual total precipitation in the four seasons (color shadings) are overlain by contours denoting 700-hPa geopotential heights. Precipitation and geopotential height climatology is derived from daily gridded data during 1997–2016 in the CDFM database^42,43^. The figure is plotted using the NCAR Command Language (Version 6.6.2) [Software]. (2019). (Boulder, Colorado: UCAR/NCAR/CISL/TDD. https://dx.doi.org/10.5065/D6WD3XH5).

To study the moisture trajectories to different parts of the TP, it is essential to have a general picture of precipitation seasonality and identify unique precipitation mechanisms in different regions with the seasons. Three features are noteworthy regarding the ratios of the seasonal to annual precipitation totals over the TP and its surroundings (Fig. 3): (1) a large area of the region witnesses the highest seasonal precipitation ratio during summer, probably associated with the high air temperature and hence high humidity during boreal summer; (2) the predominance of precipitation seasonality changes with time and location, with over 50% of the annual precipitation occurring in the southern TP and Indian Peninsula during JJA and in the northwestern part of the study region during DJF; and (3) two seasonal precipitation centres are highlighted during MAM, one in the western part and the other in the southeastern part of the study region, both aligned with the northward diversion of the quasi-zonal geopotential heights at 700- hPa and suggesting orographically induced precipitation associated with potential vorticity.

Additionally, note the accompanying wind circulation as featuring clear diversion of the 700-hPa geopotential heights northward, which suggests the likelihood for oceanic evaporated vapor to flow over the low-lying river valleys and mountain passes onto the southeastern corner of the TP throughout the year. Slight differences, however, are observed in the northward intrusion of the wind circulation, as diversion during JJA is accompanied by the summer monsoon evolution, while diversion in the other seasons is accompanied by the prevailing westerlies, thus suggesting that atmospheric water vapor during those seasons is more likely to be loaded by the westerlies rather than from the Bay of Bengal (BOB) and Indian Ocean.

### Possible moisture sources from the water vapor isotope distribution

**Figure 4:**
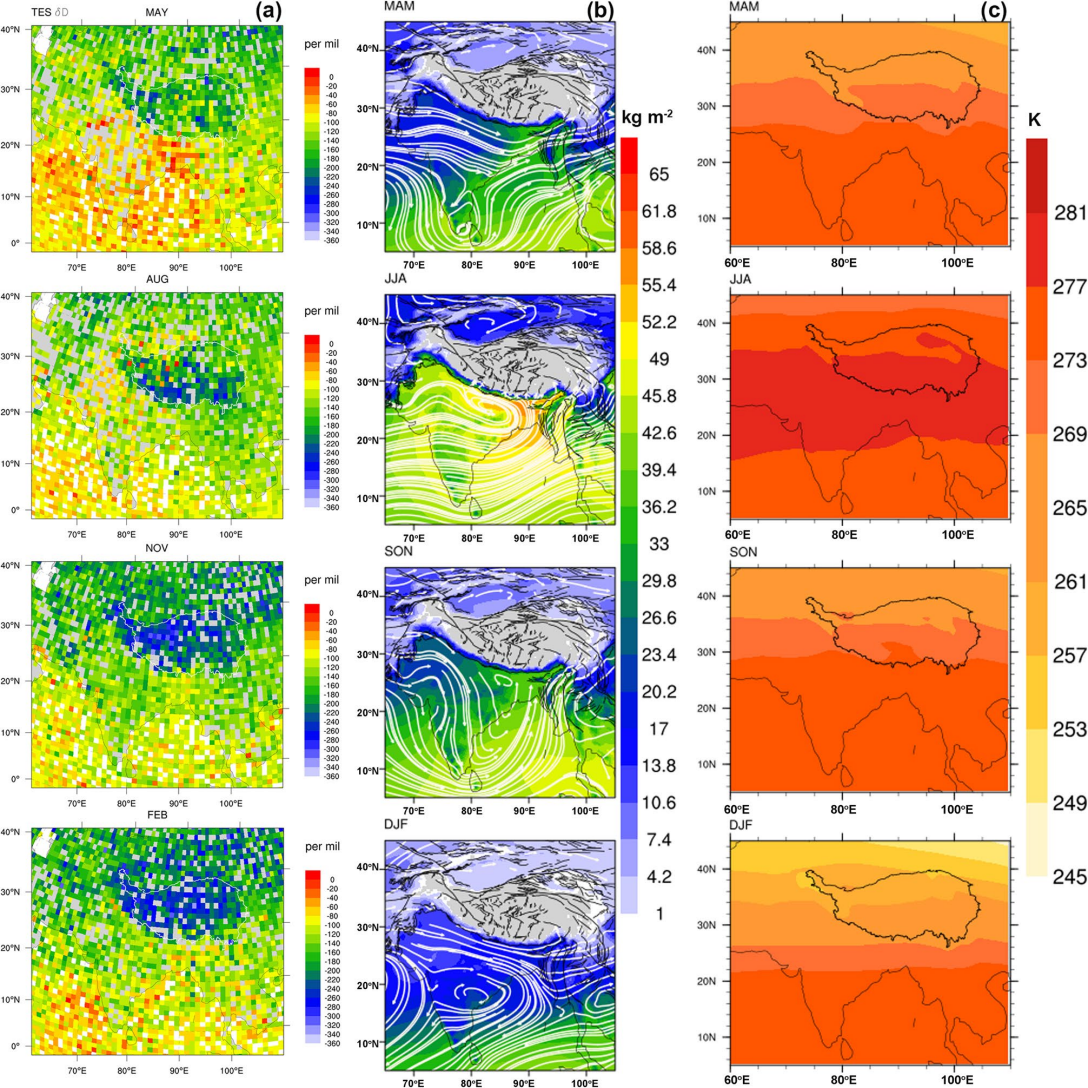
Seasonality of atmospheric water vapor isotopes and contemporary circulation and climate conditions: (**a**) selected monthly water vapor δ^2^H during an eleven-year period (September 2004 to August 2015) from TES V01,with MAY, AUG, NOV and FEB representing predominant spatial patterns in spring, summer, autumn and winter, respectively; (**b**) seasonal mean fields of precipitable water (shaded colours; calculated as the vertical integration of mixing ratio) and the zonal and meridional components (white vector) of the vertically integrated atmospheric water vapor transport fields from the surface to 600-hPa as a long-term mean seasonality derived from two decades (1999–2018) of monthly data in ERA5; and (**c**) seasonally averaged potential temperature at 600- hPa derived from seven years (2007–2013) of high-resolution daily data in the ERA-interim dataset^44^. White and black contours in (**a**) and (**c**), respectively, denote the outline of the Tibetan Plateau, while black lines in (**b**) denote major mountain ranges. The figure is plotted using the NCAR Command Language (Version 6.6.2) [Software]. (2019). (Boulder, Colorado: UCAR/NCAR/CISL/TDD. https://dx.doi.org/10.5065/D6WD3XH5).

Both δ^18^O and δ^2^H are the two most common isotopologues in water. As δ^2^H is always 8 times larger than δ^18^O in a quasi-perfect linear correlation, the potential correlation between water vapor and precipitation is hence discussed through the comparison of δ^18^O_p_ and δ^2^H in water vapor. In response to the distinct isotopic seasonality in precipitation across the TP, the isotopic composition in water vapor in the surrounding regions shows similar seasonality in its spatial pattern. With May representing MAM, it is clear to see the generally enriched isotopic compositions in the Arabian Sea, BOB and northeastern India and the southeastern corner of the TP (Fig. 4). This probably provides initial water vapor isotopic input to that in precipitation, suggesting that possible moisture sources are located to the south and southwest of the TP for the southeastern TP precipitation during MAM.

During the mature monsoon phase, as demonstrated by the August pattern, the isotopic composition in water vapor is low in the southern TP, where the water vapor mixing ratio is outstandingly high, implying a high precipitation rate and possible monsoon depletion for the southern TP. The northern TP, especially the northwestern part, features high isotopic values in water vapor and in precipitation. Corresponding to this isotopic enrichment in the northwestern TP during summer, water vapor isotopes in the Arabian Sea and Pakistan Plain prevailed at high values, suggesting possible atmospheric streams transported from the Arabian Sea northward to the northwestern TP. These findings corroborate previous studies (e.g.,^8^) demonstrating that transport by the westerlies dominates the moisture contribution in May and June, while transport by the westerlies, particularly the Indian summer monsoon and East Asian summer monsoon dominates the moisture contributions in July to September.

With November and February representative of the winter half-year, the isotopic composition in the water vapor over the TP is rather monotonic, mainly featuring much lower values than those in the surrounding areas (Fig. 4). The contrast between the spring and autumn water vapor isotopic compositions is interesting, as despite the similar potential temperature distribution, both seasons correspond to distinct isotopic compositions in precipitation (Fig. 4). This implies a direct connection between water vapor and precipitation isotopes and suggests the significance of local recycling to seasonal precipitation. Vertically integrated water vapor content to the 600-hPa pressure level shows a generally more humid environment during SON than that in MAM over the eastern and northeastern Indian Peninsula, as well as the BOB. Superposed on such a distribution of water vapor, water vapor transport shows clear differences, with MAM featuring diversion of the westerlies to the southeast along the mountain ranges in the southeastern corner of the TP, while SON features strong northeastern moisture transport from the BOB into the eastern and southeastern sections of the TP (Fig. 4b). Thus, as the moisture source is likely dominated by continental recycling over the northeastern Indian Peninsula and by moisture trajectories following the regional topography, precipitation is mainly contributed by local convection, leading to high isotopic composition in precipitation in the southeastern TP. Otherwise, the long transport distance of marine evaporation onto the southeastern TP will deplete the isotopic composition, resulting in depleted isotopic composition in both the precipitation and water vapor.

During DJF, however, even if the north- and north-westward diversion of the prevailing westerlies is frequent, the overall low water vapor content along the moisture transport trajectory implies deficient moisture sources for precipitation. The fact that the contemporary isotopic composition in precipitation is generally low is mainly attributed to low condensation temperatures. Several causes might be responsible for the isotopic seasonality in precipitation across the TP, such as the comparative location of moisture sources to sinks and transport trajectories, and the condensation temperature.

### Verification of large-scale atmospheric circulation on localized precipitation from stable water isotopes

**Figure 5:**
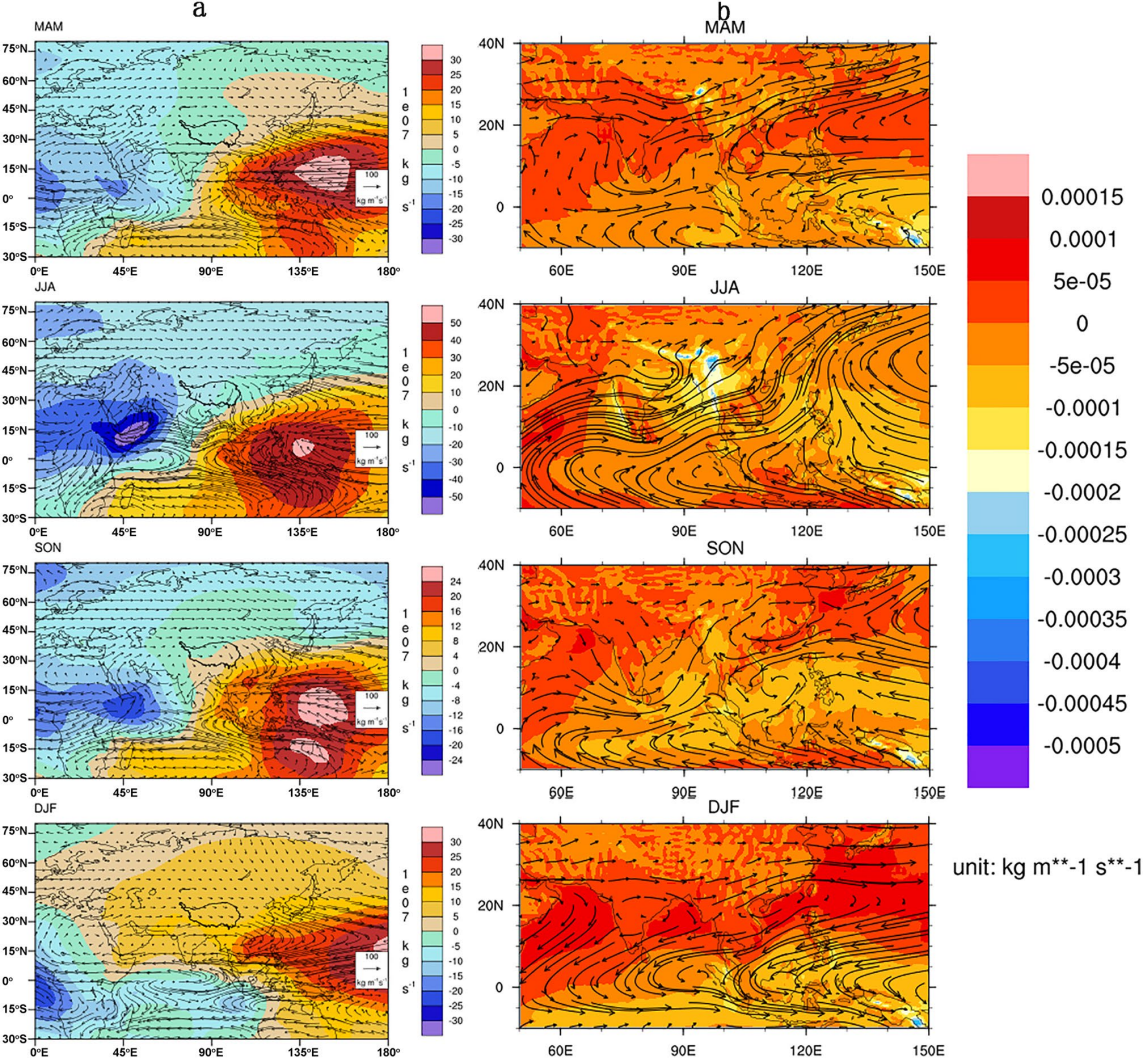
Seasonality of large-scale atmospheric circulation and vertically integrated water vapor transport. (**a**) water vapor integrated vorticity (contour; 10^7^ kg s^−1^) and rotational wind components (vector; 100 kg m^−1^ s^−1^) and (**b**) vertically integrated water vapor divergence (vector; kg m^−1^ s^−1^) and flux (shading) based on 12-year climatology (2007–2018) of vertically integrated water vapor transport from ERA5. The contour in (**a**) highlighted the TP domain. The vectors aid in the visualization of the transport pattern and intensity variation, and shaded areas show the potentials suggestive of the source (evaporation-precpitation > 0) and sink (E-P < 0) of water vapor. The figure is plotted using the NCAR Command Language (Version 6.6.2) [Software]. (2019). (Boulder, Colorado: UCAR/NCAR/CISL/TDD. https://dx.doi.org/10.5065/D6WD3XH5).

In conjunction with the atmospheric circulation, the stream function and potential for the stationary modes (seasonal mean) are examined to illustrate water vapor transport. As the stream function of water vapor flux can aid in visualizing the transport pattern and variation in intensity^45^, it shows anticyclone cells over the equatorial central Indian Ocean during MAM (Fig. 5a), suggestive of possible moisture transport from the warm ocean onto the northeastern Indian Peninsula and further to the southeastern TP. Otherwise, the approximate water balance equation indicates the Laplacian of standing potential as equivalent to the source or sink of water vapor (i.e. the difference between evaporation and precipitation). The southeastern TP shows stark contrast with other parts of the TP and features higher evaporation than precipitation. The divergence field of vertically integrated water vapor during MAM highlights that the water vapor converges over the southeastern and eastern TP, which is conducive to the maintenance of the high water vapor content over the southeastern TP during months from March through May. We acknowledge that fact that atmospheric circulation patterns do not necessarily tell the correct information on moisture sources, thus also show vertically integrated water vapor flux and divergence (Fig. 5b) to demonstrate the water content changes along the transport path for each season.

The global scenario of the convergence of water vapor flux by the stationary divergent mode reveals that the water vapor converges towards the southeastern TP during MAM, with the convergence intensifying during JJA and weakening during SON before disappearing during DJF (Fig. 5a). Correspondingly, vertically integrated water vapor transport features westerlies in MAM, turns to southwesterly during JJA and SON, but returns to westerlies in DJF, with water content water vapor flux highlighting a vapor sink in the TP in general, particularly in the southeastern corner, during MAM and JJA, which clearly weaken during SON and DJF, and even turned to be vapor source in some sporadic site across the TP (Fig. 5b). This seasonality indicates that the local Hadley circulation and summer monsoon drive water vapor transport to the southeastern TP and result in a high water vapor content there (e.g.,^8^).

The relatively low isotopic values in the southern TP during July–October have been addressed in many earlier publications, can be attributed to upstream convective activity along the moisture transport trajectory^36^ and the rainout depletion with towering clouds and strong downdrafts during precipitation^46,47^. Seasonality in the northern TP forms a stark contrast to that in the southern part, featuring low values in winter and high values in summer. As the ITC retreats to the south to the equator during boreal winter, the TP is left to the prevalence of mid-latitude westerlies resultant in dry and cold climate^10^.The sporadic vapor source suggested by the vertical integrated water vapor flux might be associated with high evaporation and prevailing westerly transport (Fig. 5), which further implies advection as the major precipitation mechanism. The low isotopic composition in precipitation is thus attributable to low air temperature during boreal winter. In fact, isotopic seasonality in the northwestern TP shows a simple feature that is in phase with general air temperature, suggesting a temperature effect under equilibrium Rayleigh fractionation. The good temperature effect on isotopic composition in the northern TP is not only significant at a particular station^24,31,48^, but also existent in a group of stations^30^.

## Conclusions and implications

This study integrated isotopic composition in precipitation at 50 stations whose locations range across the Tibetan Plateau and its surroundings, with time coverage ranging from one to 30 years. Corresponding to the spatial distribution of precipitation seasonality, featuring predominant summer precipitation over the southern TP and Indian Peninsula, significant winter precipitation dominance over the northwestern TP, and relatively even precipitation ratios in the four seasons in the western and southeastern TP, the seasonality of δ^18^O_p_ calculated for each station across the study period shows distinct heterogeneity across the TP. Specifically, the isotopic composition in precipitation shows contrasting features during non-winter months, generally featuring south-high-north-low in MAM and south-low-north-high during JJA and SON. The distinct heterogeneity in seasonality is consistent with previous studies showing a good temperature effect in the northern TP^30^. The low δ^18^O_p_ in summer and high in winter for the southern TP precipitation is consistent with a previous study of GNIP stations in tropical areas^21,49^, and can similarly be attributed to strong convective circulation associated with intertropical convergence. The unique feature of isotopic enrichment during spring in the region is noteworthy and might be associated with the northward diversion of the prevailing southwesterly still dominating in May, thus bringing oceanic evaporation from the BOB nearby and resulting in high isotopic values due to the short transport trajectory. The universal depletion in precipitation isotopes across the TP during DJF is accompanied by low convergence and prevailing water vapor transport from the west, thus indicative of advective circulation and westerly prevalence throughout the TP during non-monsoon seasons.

For the potential effect of water vapor isotopes on precipitation isotopes, it was found that the two show high spatial consistency in the presentation of the stationary (seasonal) mode. The high isotopic composition in atmospheric water vapor in the northeastern Indian Peninsula and neighbouring oceans during May verifies the southward and nearby location of the moisture source for spring precipitation over the southeastern TP. The study also shows that local Hadley and summer monsoon circulations coexist to impact water vapor convergence and transport over the TP, with topographic forcing as essential for precipitation formation and δ^18^O_p_ heterogeneity.

The heterogeneity of isotopic seasonality across the TP was first studied in this comprehensive research, revealing the close linkage between precipitation and water vapor isotopic compositions. The analysis of contemporary water vapor transport and potential help confirm the different mechanisms affecting precipitation isotopic compositions in different parts of the TP, as the southern TP is more closely related to large-scale atmospheric circulation such as local Hadley and summer monsoon circulations during seasons other than winter, and the northern TP is subject to the westerly prevalence and advective moisture supply and precipitation processes.

Yao et al.^30^ showed that isotopic compositions across the TP can be grouped into three modes, with south of 30°N as a distinct monsoon domain and north of 35°N as a distinct westerly domain. Many subsequent studies also suggested that the interplay between the westerlies and monsoons plays an important role in the spatial distribution of glaciers^3^, lakes^50^ and plant phenology^51^. According to a recent study, the warming rate on the TP is double the global average. Therefore, how such amplified warming affects the westerlies-monsoon interplay has a close linkage to water tower security. With precipitation as a direct reactor to climatic changes and its isotopic composition as a carrier of atmospheric circulation information, this topic will be further pursued in our later research.

## Methods

### Calculation of seasonal δ^18^O

Approximately 90% of the stations have contemporary amount values available; thus their monthly δ^18^O values are calculated as precipitation-weighted means, leaving the monthly δ^18^O means in the remaining stations supplemented by simple means. Pearson correlation analyses of the weighted and simple δ^18^O means for all amounts-available stations show high dependence and the same distribution at 0.01 significance level, implying little effect of this mixture on the time-series variation in δ^18^O in all the stations under study. These monthly data are further categorized into four seasons, with months from March to May as spring, from June to August as summer, from September to November as autumn, and from December to February as winter, where either the amount-weighted or simple means are calculated, depending on the amounts available, for seasonal isotopic variations.

### Precipitation data

To study the possible effect of precipitation mechanisms on its isotopic composition, high-resolution precipitation data are obtained from the High Asia Reanalysis (HAR; ftp://www.klima-ds.tu-berlin.de/NCDF4/d10km/d/2d/), including the quantity of cumulus and non-cumulus precipitation. Daily precipitation and geopotential height on the TP are downloaded from the China Meteorological Forcing Dataset (1979–2018) provided by the National Tibetan Plateau Data Center (https://data.tpdc.ac.cn). The spatial resolution was 0.1° × 0.1°.

### Vertically integrated water vapor divergence

This study used European Centre for Medium-Range Weather Forecasts (ECMWF) reanalysis ERA5 (Copernicus Climate Change Service (C3S) (2017): ERA5: Fifth generation of ECMWF atmospheric reanalyses of the global climate); Copernicus Climate Change Service Climate Data Store) offers high-resolution (0.25° × 0.25°) reanalysis data for the presentation of seasonal atmospheric circulation fields, including zonal and meridional winds, potential temperature, sea surface temperature and vertical velocity at 500-hPa, as well as vertically integrated water vapor in the zonal and meridional components.

### Calculation of water vapor isotopes from TES

The TES lite products are meant to facilitate the use of TES data. HDO and H_2_O products are obtained to allow full mapping of the isotopic composition in the atmospheric water vapor in an expansive region beyond the Tibetan Plateau. To better represent climate seasonality, eleven-year (September 2004-August 2015) data are accessed from TES V01. Qualified data for further analyses are first selected as their “SpeciesRetrievalQuality” equals 1 from each monthly dataset. The HDO and H_2_O are then extracted from the HDO_H_2_O combination in the retrieval as the first and second half of all extracted level-layers, respectively, and the vertical profile across all those levels are summed as heavy and light water isotope ratios in the atmosphere. A calibration of HDO (HDc) is conducted as HDc = HDO × (1 + 0.05) according to the empirical study^52^, and then divided by the vertically integrated sum of the lighter water vapor isotope content for δ^2^H. The δ^2^H values thus calculated are then bin-summed to a global map of 1.0° × 1.0° in spatial resolution. The satellite retrieval is later interpolated to a 0.5° resolution weighted by the cosine of the latitudes, and the monthly distribution is finally presented in a geographically limited orthographic projection map.

### Streamfunction and vorticity

To illustrate the relationship between atmospheric circulation and water vapor transport, the streamfunction and potential are calculated. The streamfunction and the potentials and their vectors are calculated following^45^, with the potential field obtained by solving the Poisson equation using the convergence of water vapor transport flux as forcing, while the streamfunction is calculated as the Laplacian of vorticity. In practice, Fig. 5 is plotted using NCL, with the vorticity calculated using the ilapsG-Wrap function from divergence, which is derived using the uv2dvG_Wrap function, while the rotational wind components are calculated using the ur2uvG_Wrap function from the vorticity, which is derived using the uv2vrG_Wrap function.

## Data Availability

The reanalysis data that support the findings of this study are available from High Asia Reanalysis (HAR; ftp://www.klima-ds.tu-berlin.de/NCDF4/d10km/d/2d/) and the fifth generation of atmospheric reanalyses of the global climate of the ECMWF (https://cds.climate.copernicus.eu/), including zonal and meridional winds, temperature and geopotential heights, as well as the vertical integral of water vapor flux and divergence. The China meteorological forcing data are provided by the National Tibetan Plateau Data Center (https://data.tpdc.ac.cn). The TES Lite data are provided by the Atmospheric Science Data Center at the NASA Langley Research Center through the website: https://eosweb.larc.nasa.gov/project/tes/. The stable isotope data in precipitation newly presented in this study are available from the corresponding author upon reasonable request.
